# Metformin is associated with improved clinical outcomes in patients with melanoma: a retrospective, multi-institutional study

**DOI:** 10.3389/fonc.2023.1075823

**Published:** 2023-06-16

**Authors:** Ryan C. Augustin, Ziyu Huang, Fei Ding, Shuyan Zhai, Jennifer McArdle, Anthony Santisi, Michael Davis, Cindy Sander, Diwakar Davar, John M. Kirkwood, Greg M. Delgoffe, Allison Betof Warner, Yana G. Najjar

**Affiliations:** ^1^ Department of Medicine, University of Pittsburgh, Pittsburgh, PA, United States; ^2^ UPMC Hillman Cancer Center, Pittsburgh, PA, United States; ^3^ Department of Biostatistics, UPMC Hillman Cancer Center, Pittsburgh, PA, United States; ^4^ Department of Biomedical Informatics, University of Pittsburgh, Pittsburgh, PA, United States; ^5^ Department of Immunology, University of Pittsburgh, Pittsburgh, PA, United States; ^6^ Memorial Sloan Kettering Cancer Center, New York, NY, United States

**Keywords:** tumor microenvironment (TME), melanoma, checkpoint blockade, oxidative phosphorylation, tumor infiltrating lymphocyte, progression-free survival, metformin

## Abstract

**Background:**

Pre-clinical studies have shown that metformin reduces intratumoral hypoxia, improves T-cell function, and increases sensitivity to PD-1 blockade, and metformin exposure has been associated with improved clinical outcomes in various types of cancer. However, the impact of this drug in diabetic melanoma patients has not yet been fully elucidated.

**Methods:**

We reviewed 4,790 diabetic patients with stage I-IV cutaneous melanoma treated at the UPMC-Hillman Cancer Center and Memorial Sloan Kettering Cancer Center between 1996-2020. The primary endpoints included recurrence rates, progression free survival (PFS), and overall survival (OS) with and without metformin exposure. Tabulated variables included BRAF mutational status, immunotherapy (IMT) by type, and incidence of brain metastases.

**Results:**

The five-year incidence of recurrence in stage I/II patients was significantly reduced with metformin exposure (32.3% vs 47.7%, p=0.012). The five-year recurrence rate for stage III patients was also significantly reduced (58.3% vs 77.3%, p=0.013) in the metformin cohort. OS was numerically increased in nearly all stages exposed to metformin, though this did not reach statistical significance. The incidence of brain metastases was significantly lower in the metformin cohort (8.9% vs 14.6%, p=0.039).

**Conclusion:**

This is the first study to demonstrate significantly improved clinical outcomes in diabetic melanoma patients exposed to metformin. Overall, these results provide further rationale for ongoing clinical trials studying the potential augmentation of checkpoint blockade with metformin in advanced melanoma.

## Background

Melanoma is the fifth most common cancer diagnosed in the United States, with an incidence that continues to rise (1). Most patients have localized disease with excellent survival outcomes; however, five-year survival rates dramatically decrease for patients with locoregionally advanced or metastatic disease (1). The current era of immunotherapy (IMT) has revolutionized the treatment of advanced melanoma in both the adjuvant and metastatic setting (2–5), though validated predictive biomarkers are lacking to date (6). Metformin, a commonly utilized type II diabetes drug, has been shown to metabolically reprogram the tumor microenvironment (TME) (7), and in pre-clinical models, to augment the effectiveness of anti-programmed cell death protein 1 (PD1) IMT (8). The purpose of this study is to examine the impact of metformin on the clinical outcomes of diabetic patients with melanoma.

Several retrospective studies of various cancer types have demonstrated improved clinical outcomes in patients taking metformin (9). An analysis of over 300 diabetic patients with endometrial cancer revealed improved progression free survival (PFS) (HR 0.59, p=0.01) and overall survival (OS) (HR 0.43, p=0.005) in patients taking metformin versus those not exposed to the drug (10). A study of 302 diabetic patients with pancreatic cancer also demonstrated improved OS when comparing patients with and without metformin exposure (HR 0.68, p=0.003) (11). A 2018 retrospective study of 55 patients with unresectable stage IIIC and stage IV melanoma patients treated with IMT showed improved overall response rate (ORR) and PFS in patients exposed to metformin (12), though these results were not statistically significant. Furthermore, this study only included patients with advanced stage disease, and compared diabetic patients treated with metformin with non-diabetic patients who were thus not exposed to metformin. We therefore sought to analyze a large cohort of diabetic melanoma patients across all stages of disease to assess the association between metformin exposure and recurrence rates, PFS, OS, and incidence of brain metastases, among other variables.

A variety of mechanisms have been proposed to explain the improved clinical outcomes in cancer patients with metformin exposure. Many of these hypotheses center around metabolic alterations of the TME, which is understood to have important implications for tumor infiltrating lymphocytes (TIL), and thus, clinical outcomes with IMT (13). Metformin has been shown to activate a variety of both AMP-activated protein kinase (AMPK)-dependent and independent cellular signaling pathways that may alter the metabolic milieu of the TME and corresponding T-cell function (14). Metformin alters pro-inflammatory cytokine signaling in the TME, thereby rescuing exhausted CD8+ TILs and promoting anti-tumor effects (7, 15). Additionally, metformin was found to inhibit PD-L1 signaling *via* endoplasmic reticulum associated degradation; an effectual checkpoint blockade further enhancing TIL function (16). Metformin-mediated metabolic shifts have also been shown to inhibit regulatory T cells (Treg) in the TME and may also enhance immunity *via* alteration in the microbiome (17, 18). Furthermore, the association between metformin, a known complex I inhibitor, and hypoxia reversal in the TME is of significant interest, as it is now understood that the impact of hypoxia on immune function is largely detrimental. Hypoxia can induce an immunosuppressive state *via* enhanced Treg function and inhibitory T-cell receptor signaling (19). Hypoxic signaling can also lead to TIL dysfunction and phenotypic exhaustion, with important clinical implications for immune-based therapy (20, 21).

Notably, the related and previously studied biguanide, phenformin, has been shown to be a more potent mitochondrial inhibitor as compared to metformin (22). While both biguanides have a similar mechanism of action related to AMPK activation and oxidative phosphorylation (ox-phos) inhibition, phenformin has been shown to reduce tumorigenesis to a greater degree in some pre-clinical murine models (23). Additional pre-clinical work suggests that phenformin mediated AMPK activation can directly inhibit the mitogen-activated protein kinase (MAPK) pathway and provide a synergistic effect in combination with BRAF/MEK inhibitors in melanoma tumors with BRAF V600E/K mutations (24). Given these findings, an ongoing phase I clinical trial (NCT03026517) aims to assess the safety and efficacy of phenformin plus BRAF and MEK inhibitors in patients with BRAF mutant advanced melanoma. Apart from this trial, phenformin has been largely withdrawn from clinical use based on prior reports linking phenformin with higher rates of lactic acidosis and thus was not included in this retrospective study.

We and others have reported that the metabolic landscape of the TME is innately immunosuppressive (25, 26). Deregulated metabolism of tumor cells results in both a lack of nutrients such as glucose and oxygen, and buildup of toxic byproducts such as lactic acid. We previously demonstrated that melanoma patient tumor cells can be metabolically profiled directly from biopsies, and that deregulation of metabolism in tumor cells reveals insight into the status of the antitumor immune response to checkpoint blockade (27). Specifically, we showed that oxidative tumor cell metabolism is linked to resistance to anti-PD1 IMT; TIL isolated from melanoma tumors with high oxidative metabolism are more exhausted and less functional. High oxidative metabolism in tumor cells and the consequent generation of tumor hypoxia is associated with resistance to anti-PD1 and worse clinical outcomes, including decreased PFS, duration of response (DOR), and OS (27). We therefore developed a murine melanoma model to evaluate the impact of complex I inhibition on intra-tumoral hypoxia and T-cell function (27). Tumors from mice with complex I knock-down showed reduced ox-phos, improved T cell function, and decreased T cell exhaustion. Furthermore, only the complex I knock-down models showed response to anti-PD1, whereas GLUT1 knock down did not impact responses to IMT, suggesting that oxidative metabolism is implicated in resistance to anti-PD1 based IMT (27). Given these findings, we sought to evaluate actual clinical outcomes *via* this large retrospective analysis of diabetic melanoma patients with and without metformin exposure.

## Study methods

**Figure 1:**
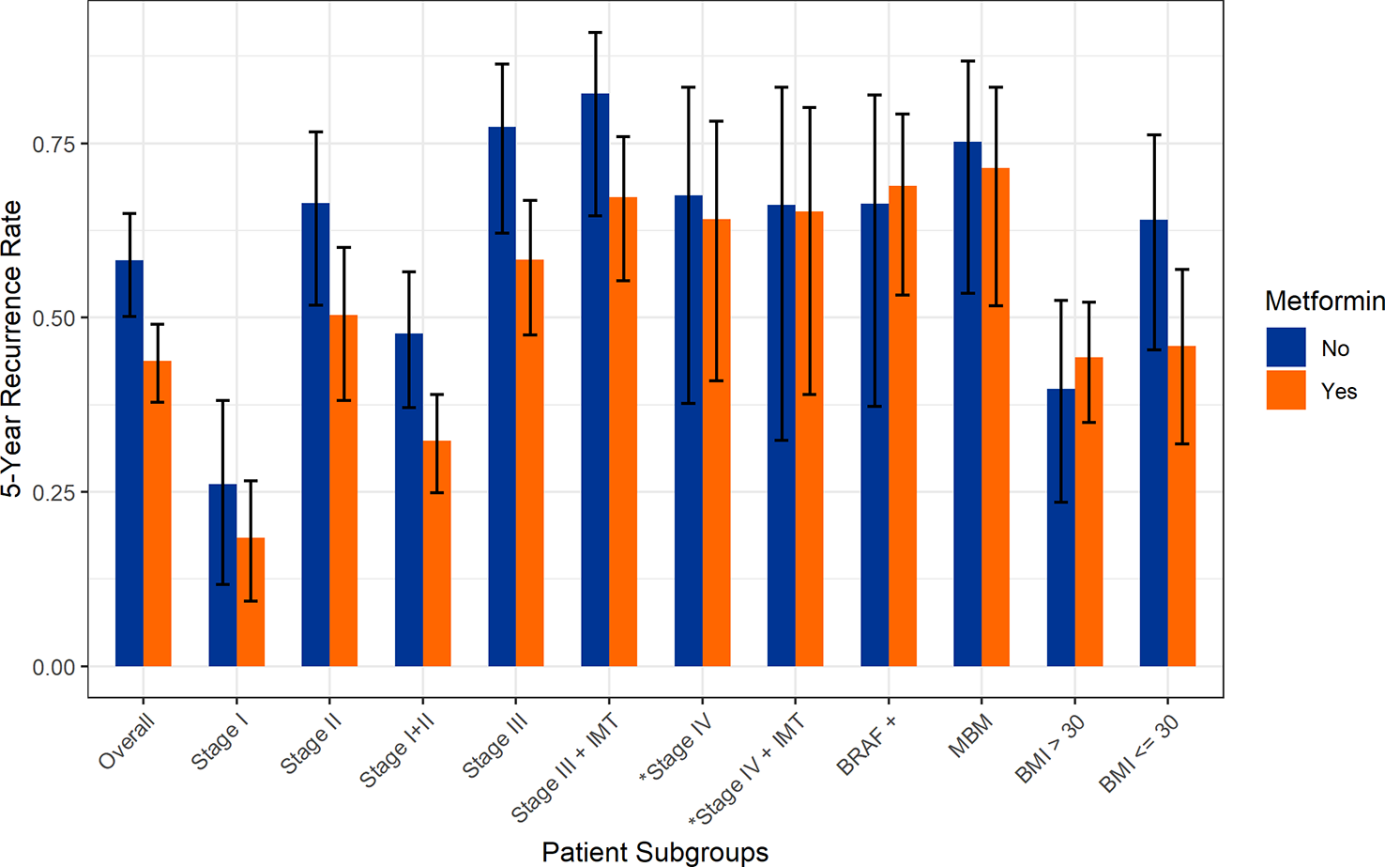
Five-year recurrence rates stratified by stage and metformin exposure. *Rates of disease progression.

A retrospective cohort study was conducted at the UPMC-Hillman Cancer Center (HCC) and Memorial Sloan Kettering Cancer Center (MSKCC). With IRB approval from each institution, 4,790 charts were reviewed from patients seen between 1996-2020. Relevant charts were identified based on coding for the diagnoses of cutaneous melanoma and type 2 diabetes (T2DM). After initial review, 668 patients were found to have T2DM, with stage identified at initial diagnosis (**Figure 1**). This final subset was further categorized into metformin versus no metformin exposure and the key variables were tabulated for each patient, including: stage at initial diagnosis (AJCC 7 criteria), age at diagnosis, sex, BMI, performance status (Eastern Cooperative Oncology Group, ECOG), ulceration, BRAF mutational status, metformin exposure (yes/no), metformin dose, recurrence status, time to recurrence, use of IMT and indication (adjuvant/systemic), type of IMT (high dose interferon alpha-2b (HDI), high-dose IL-2, anti-PD1, anti-CTLA4, anti-PD1 plus anti-CTLA4), presence of brain metastases, date of last follow up and vital status. Histologic subtype was also collected but consistent data was not available in a large portion of our early patient records. Nondiabetic patients were not included in order to reduce confounding variables between metformin exposed and non-exposed patients.

The primary objectives of this study included recurrence rates with and without metformin in patients with stage I-III melanoma, and PFS and OS in stage I-IV patients (with and without metformin). Secondary outcomes included recurrence and survival outcomes stratified by BMI, IMT treatment, and presence of brain metastases with or without metformin exposure. Recurrence rates were defined as the proportion of patients with documented recurrence (stage I-III) or disease progression (PD) (stage IV). PFS was defined as time in months from the diagnosis of melanoma to PD or death or being censored at the last follow-up. OS was defined as time in months from the diagnosis of melanoma to death or being censored at the last follow-up.

Comparison of recurrence and survival rates was performed using the Cox proportional hazards model. Normal continuous variables were tested using the t-test. Non-normal continuous variables were tested using the Wilcoxon rank-sum test. Categorical variables were tested using the Chi-squared test, with Fisher’s exact test being used for small counts. Crude recurrence rates between metformin groups were compared using the Fisher’s exact test. All analyses were performed using R version 4.1.1.

## Results

### Baseline characteristics

Patient demographics (n=668) are listed in **Table 1**. In total, 422 patients were treated with metformin and 246 patients were not. The mean age of the entire cohort was 64.2 years, and 68.2% were male. At initial diagnosis, 210 patients had stage I, 183 stage II, 195 stage III, and 80 patients had stage IV disease (AJCC 7). BRAFV600E/K mutation was found in 33.6% of the entire cohort, and this was not significantly different between the two groups. Most patients had an ECOG performance status of 0 or 1, and there was a higher proportion of ECOG 0 patients in the metformin cohort (55.8% vs 37.9%, p=0.001). Ulceration was present in 34.3% (n=169) of patients at diagnosis; this was not significantly different between the two groups. LDH levels at diagnosis were similar between the cohorts.

**Table 1:** Demographics and baseline characteristics.

	Total n=668	Metformin n=422	No Metformin n=246	p-value
Age at diagnosis (years)				
**Mean(Sd)**	64.2(12.3)	63.9(11.74)	64.7(13.2)	0.32
Gender, n(%)				
**M**	455(68.2)	286(67.9)	169(69.6)	0.91
**F**	212(31.8)	70(31.2)	77(30.4)	
BMI at diagnosis, median(IQR)				
	31.6(28, 36.3)	31.65 (28.0, 37.1)	31.1 (27.8, 35.7)	0.22
Stage at diagnosis, n				
**I**	210	138	72	0.33
**II**	183	109	74	0.24
**III**	195	129	66	0.33
**IV**	80	46	34	0.27
LDH at diagnosis, >ULN(%)				
	48.4	44.0	58.3	0.139
ECOG Performance Status, n(%)				
**0**	207(49.3)	149(55.8)	58(37.9)	**0.001**
**1**	187(44.5)	106(39.7)	81(52.9)	**0.03**
**2**	26(6.2)	12(4.5)	14(9.2)	0.08
Brain metastases, n(%)				
**Yes**	68(11.0)	35(8.9)	33(14.6)	**0.039**
**No**	552(89.0)	359(91.1)	193(85.4)	
Ulceration, n(%)				
**Ulcerated**	169(25.3)	108(25.6)	61(24.8)	0.07
**Not ulcerated**	323(48.4)	215(50.9)	108(43.9)	
**NA**	176(26.3)	99(23.5)	77(31.3)	
Number of metastases, n(%)				
**0**	189(39.3)	129(41.9)	60(34.7)	0.062
**1**	66(13.7)	49(15.9)	17(9.8)	0.059
**2 or 2+**	48(10.0)	29(9.4)	19(11.0)	0.76
**3 or 3+**	100(20.8)	56(18.2)	44(25.4)	0.12
**4 or 5**	27(5.6)	13(4.2)	14(8.1)	0.11
**5+**	51(10.6)	32(10.4)	19(11.0)	0.9
Prior adjuvant, n(%)				
**Yes**	112(36.7)	78(42.2)	34(28.3)	0.06
**No**	163(53.4)	95(51.4)	68(56.7)	
Type of adjuvant, n(%)				
**HDI**	60(53.6)	42(53.8)	18(52.9)	0.9
**anti-PD1**	32(28.6)	23(29.5)	9(26.5)	0.82
**anti-CTLA4**	5(4.5)	4(5.1)	1(2.9)	0.9
**Other**	15(13.4)	9(11.5)	6(17.6)	0.4
BRAF V600E/K mutations, n(%)				
**Yes**	86(33.6)	60(37.0)	26(27.7)	0.163
**No**	170(66.4)	102(63.0)	68(72.3)	
Metformin median dose				
**Median(median IQR)**		1000 mg daily (1000-2000)		
Systemic Therapy, n(%)				
**Yes**	350(52.4)	226(53.6)	124(50.4)	0.42
**No**	156(23.4)	94(22.2)	61(24.8)	
Type of therapy, n(%)				
**anti-CTLA4**	44(12.6)	27(11.9)	17(13.7)	0.62
**anti-PD1**	100(28.6)	69(17.3)	31(25)	0.33
**anti-CTLA4/anti-PD1**	70(20)	45(20)	25(120.2)	0.9
**HDI**	108(30.9)	76(33.6)	42(33.9)	0.10
**anti-TIM3**	1(0.3)	0(0)	1(0.8)	–
**IL-2**	5(1.4)	2(0.9)	3(2.4)	-
**Oncolytic viral therapy**	10(2.9)	5(2.2)	5(4.0)	–

Adjuvant therapy was reported in 36.7% (n=112) of eligible patients; this was similar between the two groups, with HDI being most common. 52.4% (n=350) of patients were exposed to IMT, with the most common being HDI (n=108), anti-PD1 therapy (n=100), and anti-CTLA4 (n=44); this was similar between the metformin and no metformin groups.

The median metformin dose was 1000 mg daily (range 250-2000 mg; dose reported in 269 of 422 patients). Of 436 patients with BMI data, 39.7% were obese (BMI >30) with 60.3% non-obese (BMI ≤30). Other variables including years since T2DM diagnosis, mean Hgb A1C, utilization of other hypoglycemic agents, and duration of metformin therapy were not consistently available.

### Recurrence rates

For the overall cohort (n=668), the five-year incidence of recurrence was significantly lower in patients exposed to metformin (43.8% vs 58.2, p=0.002). In a pooled cohort of patients with stage I or II melanoma (n=393), we again note that the five-year incidence of recurrence was significantly lower in patients exposed to metformin (32.3% vs 47.7%, p=0.012); this was still significant after adjusting for age, gender, and BMI (**Figure 1** and **Table 2**). Evaluating individual stage cohorts for stage I and II, we find a consistent numerical increase in 5-year recurrence rates for patients not exposed to metformin, though this did not reach statistical significance (**Table 2**).

**Table 2:** Recurrence rates stratified by stage/subgroup and metformin exposure.

Time to recurrence	Stratification	Metformin	No Metformin	p-value
Crude	Overall	42.4% (37.8%, 47.2%)	55.3% (49%, 61.4%)	**0.002**
	Stage I and II	32% (26.5%, 38.1%)	49.3% (41.3%, 57.4%)	**0.001**
	Stage I	18.8% (13.2%, 26.2%)	31.9% (22.2%, 43.5%)	**0.04**
	Stage II	48.6% (39.4%, 57.9%)	66.2% (54.8%, 76%)	**0.023**
	Stage III	58.9% (50.2%, 67.1%)	71.2% (59.2%, 80.8%)	0.117
	Stage III with IMT	69.7% (60%, 77.9%)	84.1% (70.2%, 92.2%)	*0.097*
	BRAF+	80% (68%, 88.3%)	76.9% (57.2%, 89.2%)	0.777
	BMI>30	37.1% (30.6%, 44.1%)	34.7% (24.7%, 46.4%)	0.775
	BMI<=30	38.4% (29.9%, 47.7%)	56.9% (44%, 68.9%)	**0.024**
5-Year	Overall	43.8% (37.9%, 49.1%)	58.2% (50.2%, 65%)	**0.002**
	Stage I and II	32.3% (24.9%, 39%)	47.7% (37.1%, 56.6%)	**0.012**
	Stage I	18.4% (9.4%, 26.6%)	26.1% (11.7%, 38.2%)	0.338
	Stage II	50.3% (38.1%, 60.1%)	66.4% (51.8%, 76.6%)	0.053
	Stage III	58.3% (47.6%, 66.8%)	77.3% (62.1%, 86.4%)	**0.013**
	Stage III with IMT	67.3% (55.3%, 76%)	82.1% (64.6%, 91%)	*0.067*
	BRAF +	68.9% (53.3%, 79.2%)	66.4% (37.3%, 81.9%)	0.84
	BMI > 30	44.3% (35%, 52.2%)	39.7% (23.5%, 52.5%)	0.597
	BMI <= 30	45.9% (31.9%, 57%)	64% (45.4%, 76.3%)	0.068

Similarly, the five-year recurrence rate in all stage III patients (n=195) was lower with metformin compared to no metformin exposure (58.3% vs 77.3%, p=0.013) (**Table 2**). In patients treated with adjuvant therapy (stage IIB-IIIC, n=112), the recurrence rate was lower 59.3% vs 67.6% with metformin exposure, though this did not reach statistical significance (p=0.42). The overall incidence of brain metastases (MBM) was significantly lower in the metformin cohort (8.9% vs 14.6%, p=0.039) (**Table 1**), though significant changes in survival were not seen in the MBM subgroup. A low BMI (<30) was marginally protective against recurrence in the metformin group (45.9% vs 64.0%, p=0.068) (**Table 2**). Regardless of metformin exposure, BRAF mutation was not associated with any difference in recurrence. Additionally, higher BMI was associated with a nonsignificant reduction in recurrence regardless of metformin status (36.5% vs 44.7%, p=0.089).

### Survival outcomes

There was no significant difference in PFS between the metformin cohorts (**Table 3** and **S1**). Patients with lower stage disease (I/II) were noted to have an OS hazard ratio of 0.563 (p=0.084) in the metformin group (**Table 4** and **S2**). When stratified by higher BMI, patients exposed to metformin had an OS hazard ratio of 0.598 (p=0.076) (**Table 4**). All survival data were adjusted for age, sex, and BMI at diagnosis. Other covariates including BRAF mutation, ulceration, performance status, and adjuvant therapy were not included in the Cox regression models due to the relatively large proportion of missing data.

**Table 3:** Progression free survival hazard ratios (HR) for each stage and subgroup by metformin exposure.

Stratification	HR (CI)	p-value
**All**	0.883 (0.64-1.22)	0.447
**I**	1.368 (0.42-4.5)	0.603
**II**	0.682 (0.39-1.19)	0.181
**I+II**	0.757 (0.46-1.24)	0.269
**III**	0.785 (0.47-1.30)	0.349
**III+IMT**	0.881 (0.49-1.60)	0.676
**IV**	1.866 (0.78-4.44)	0.159
**IV+IMT**	1.519 (0.60, 3.87)	0.381
**BRAF+** (all)	1.385 (0.68-2.82)	0.37
**MBM** (all)	1.315 (0.63-2.76)	0.468
**BMI >30** (all)	1.058 (0.67-1.68)	0.81
**BMI <30** (all)	0.76 (0.48-1.21)	0.249

**Table 4:** Overall survival hazard ratios (HR) for each stage and subgroup by metformin exposure.

Stratification	HR (CI)	p-value
**All**	0.744 (0.495-1.119)	0.156
**I**	0.53 (0.144-1.954)	0.34
**II**	0.561 (0.262-1.2)	0.136
**I+II**	0.563 (0.294-1.08)	0.084
**III**	0.907 (0.434-1.895)	0.795
**III + IMT**	0.856 (0.395-1.855)	0.69
**IV**	1.005 (0.43-2.35)	0.99
**IV + IMT**	0.723 (0.291-1.797)	0.485
**BRAF+**	1.149 (0.388-3.405)	0.802
**MBM** (all)	1.119 (0.476-2.629)	0.796
**BMI >30** (all)	0.598 (0.338-1.056)	0.076
**BMI <30** (all)	0.916 (0.507-1.654)	0.772

## Discussion

Over the past decade, IMT has led to a groundbreaking improvement in clinical outcomes of patients with advanced melanoma, with 6.5 year OS for ipilimumab plus nivolumab of 49% (95% CI 44-55%) (28). However, a significant subset of patients develop primary or secondary resistance (29). Considerable effort is underway to better understand the mechanisms of resistance to IMT. It is now well understood that immunosuppressive and metabolically hostile TME, including decreased pH, altered amino acid metabolism, mitochondrial dysfunction, and hypoxia have substantial effects on the phenotype and function of TIL (8). This altered metabolic milieu of the TME may help to explain why only half of patients ultimately benefit from checkpoint blockade.

Seahorse cell analysis has been used to quantitatively measure oxidative phosphorylation (OCR) and glycolytic metabolism (ECAR) in melanoma tumor cells, and baseline tumor cell metabolism has implications on the TME, TIL function and clinical outcomes (8). Metformin reduced OCR in B16 bearing mice, and resulted in decreased intratumoral hypoxia. Furthermore, when mice were inoculated with a PD1 resistant melanoma cell line, the combination of metformin with anti-PD1 led to tumor regression in 80% of mice, whereas metformin or anti-PD1 monotherapy showed minimal anti-tumor efficacy, suggesting a synergistic effect on T-cell function in the TME (8). We have also shown that high oxidative metabolism in patient derived melanoma tumor cells is associated with decreased function and increased exhaustion of TIL, with significantly worse clinical outcomes, suggesting that high oxidative metabolism in melanoma tumor cells is associated with resistance to anti-PD1 (27). Furthermore, an experimental ox-phos inhibitor, IACS-010759, has shown improved survival in a pre-clinical murine MBM model (30).

While these pre-clinical models show that metformin has a beneficial impact on TIL function, our present clinical data shows a notable improvement in clinical outcomes for diabetic melanoma patients taking metformin. In the metformin group, we note a significant reduction in 5-year recurrence rates (stages I-III) and rates of PD (stage IV) for patients in the overall cohort, and also for patients with stage I/II and stage III disease. The finding of a numerical increase in 5-year recurrence in patients not on metformin may not have reached statistical significance in the individual stage I and II cohorts due to a smaller sample size. The significant reduction in recurrence rates noted here for the overall cohort, and for stage I/II patients taking metformin is certainly of interest, albeit difficult to attribute to any single metabolic change that might derive from metformin’s multiple downstream signaling effects. Based on pre-clinical data (8, 27), one may hypothesize that patients with early stage melanoma taking metformin have a more favorable TME and more efficient TIL anti-tumor activity at a critically early stage of pathogenesis. A similar study of 242 diabetic gastric cancer patients with or without metformin exposure showed significantly improved survival in only the localized (N0) subgroup, promoting this hypothesis in early stage disease (31). Our study also showed decreased 5-year recurrence in patients with stage III melanoma who took metformin. Overall, these findings in the context of our pre-clinical work suggest a more concerted and targeted metabolic effect secondary to metformin exposure (e.g. reduction in tumor cell oxidative metabolism improved TIL function, and reversal of hypoxia) (8, 25), though definitive correlation would require tumor-derived biomarker assessment. The decrease in recurrence rates for metformin-exposed stage III patients treated with or without IMT was not significantly improved with IMT, potentially due to the relatively small number of patients in this subgroup. Additionally, while there was an overall trend towards improved survival across all stages, patients with metastatic disease at diagnosis (n=80) did not exhibit any difference in survival with metformin exposure, though this was relatively small cohort. Further, patients may be less likely to derive benefit from metformin at more advanced stages of disease, when the drug is less able to significantly impact the TME due to nutrient competition, restricted drug delivery, and multiple acquired mechanisms of resistance.

Metformin exposure was associated with reduced incidence of brain metastases in this study. Our data aligns with previous work demonstrating improved OS in a MBM murine model after administration of an ox-phos inhibitor (30). In correlation, a recent gene expression analysis of intracranial and extracranial melanoma metastases revealed a significant increase in ox-phos expression along with immunosuppression in melanoma brain metastases (30), suggesting a selective pressure favoring the outgrowth of highly oxidative hypermetabolic clones. Given the biochemical properties permitting metformin to cross the blood-brain barrier, high prevalence of brain metastases in melanoma, and association with increased morbidity and mortality, these studies provide grounds for ongoing translational research in the field of immunometabolism (32, 33).

Notably, our data showing significantly improved recurrence rates with metformin exposure did not correlate with survival outcomes. Given the retrospective nature of this study with some of the patient data originating from over two decades ago, there are significant confounding factors that likely affected differential survival in these patient cohorts. Namely, improvements in SOC treatments leading to improved overall survival may have over time diluted metformin-mediated survival advantages. Regardless, after adjusting for various baseline characteristics, patients with stage I-II melanoma who were exposed to metformin had reduced recurrence rates. Though tissue biomarkers were not available in this retrospective study, our prior data suggests a biological shift towards an anti-tumor phenotype of more functional TILs with critical relevance towards combatting the immunosuppressive nature of the TME (8, 25).

Hahn et al. have shown that obesity is associated with both improved survival and reduced ox-phos in metastatic melanoma (34), and we therefore assessed obesity related outcomes in our patient population. While lower BMI was marginally protective against recurrence in the metformin cohort, survival outcomes suggested the alternative. Patients with an elevated BMI and metformin exposure had a lower OS hazard ratio. Further, a nonsignificant reduction in recurrence was seen in patients with higher BMI, regardless of metformin exposure. Taken together, these data suggest reduced ox-phos, potentially *via* metabolic changes induced through obesity or metformin, could lead to improved outcomes in patients with melanoma (35).

Our retrospective study has several limitations inherent to its nature. Conclusions drawn are based on data available in the electronic medical record. However, all the patients included in this cohort were seen at regular intervals for routine follow-up, and information on demographics and clinical outcomes such as response rates, treatment utilized, and recurrence was readily available. Furthermore, data on response to therapy was based on investigator assessment, which may increase variability with regards to this outcome. Our sample size for patients with stage III treated with adjuvant immunotherapy precludes our ability to draw clear conclusions in this patient population. Similarly, our cohort size for stage IV was smaller than for patients with earlier stage melanoma, as would be expected. Most covariates were well balanced between the two cohorts. However, a significantly higher proportion of patients with an ECOG 0 or 1 were found in the metformin exposed group, a potential confounding variable. Further, more patients in the metformin cohort had received adjuvant therapy, though this did not reach statistical significance. Despite its limitations, this analysis generates a hypothesis of clinically improved outcomes in diabetic melanoma patients treated with metformin, compared to diabetic melanoma patients not exposed to this drug. This is in line with reports of metformin in other malignancies and is confirmatory of our pre-clinical findings. In addition, we screened nearly 5,000 patients for inclusion in this study, resulting in a considerable cohort size of diabetic melanoma patients treated across two tertiary medical centers.

Overall, these data provide further rationale for prospectively investigating the role of metformin in the treatment of advanced melanoma and/or prevention of recurrence in the adjuvant setting. A prospective translational trial investigating anti-PD1 with or without metformin is underway (NCT03311308) and will assess the role of decreasing tumor cell ox-phos and reversing hypoxia in the TME together with checkpoint inhibition for the treatment of advanced melanoma.

## Data availability statement

The original contributions presented in the study are included in the article/**Supplementary Material**. Further inquiries can be directed to the corresponding author.

## Ethics statement

The studies involving human participants were reviewed and approved by University of Pittsburgh IRB Committee. Written informed consent for participation was not required for this study in accordance with the national legislation and the institutional requirements.

## Author contributions

RA: Data collection, analysis, manuscript writing ZH, FD, SZ, JM, AS, MD, CS: Data collection, statistical support DD, JM, GD: Editorial support AW, YN: project design, analysis, editorial oversight. All authors contributed to the article and approved the submitted version.
