# Supplementary material for: Metformin is associated with improved clinical outcomes in patients with melanoma: a retrospective, multi-institutional study
Source: Front Oncol. 2023 Jun 16;13:1075823. doi: 10.3389/fonc.2023.1075823 (PMC10312386; doi:10.3389/fonc.2023.1075823)
Supplement: Supplementary file 1 [file DataSheet_1.pdf]

Supplementary Figures

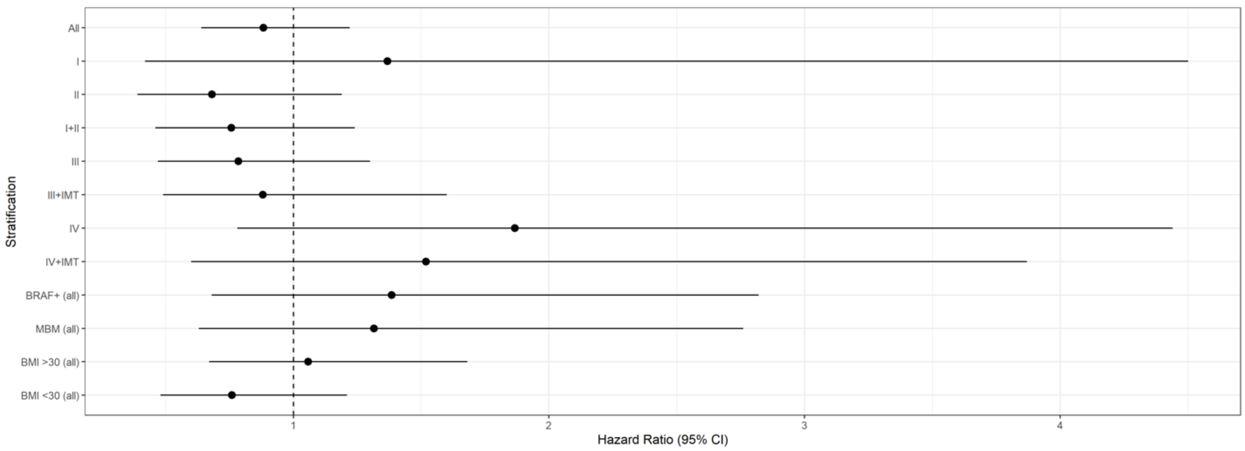

S1. Forest plot depicting progression free survival hazard ratios (HR) for each stage and subgroup by metformin exposure as shown in table 3

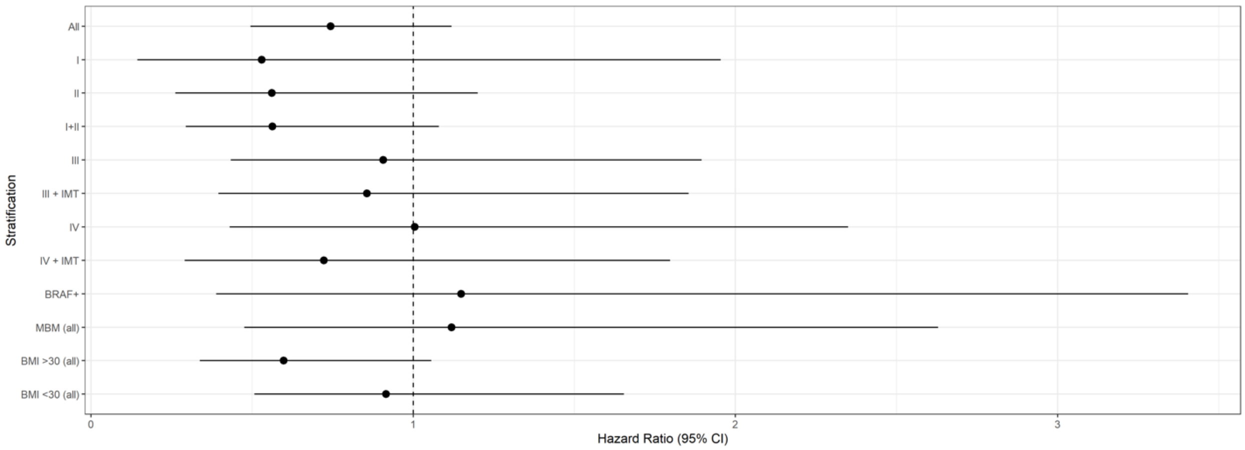

S2. Forest plot depicting overall survival hazard ratios (HR) for each stage and subgroup by metformin exposure as shown in table 4
